# p53 nuclear accumulation and ERα expression in ductal hyperplasia of breast in a cohort of 215 Chinese women

**DOI:** 10.1186/1756-9966-29-112

**Published:** 2010-08-16

**Authors:** Xiao-yun Mao, Chui-feng Fan, Hua-chuan Zheng, Jing Wei, Fan Yao, Feng Jin

**Affiliations:** 1 Department of Breast Surgery, Department of Surgical Oncology, Research Unit of General Surgery, the First Affiliated Hospital of China Medical University, Shenyang, Liaoning Province, (110001), China; 2 Department of Pathology, the First Affiliated Hospital and College of Basic Medical Sciences of China Medical University, Shenyang, (110001), China; 3 Department of Biochemistry and Molecular Biology, College of Basic Medical Sciences of China Medical University, Shenyang, (110001), China

## Abstract

**Introduction:**

Women with ductal hyperplasia including usual ductal hyperplasia (UDH) and atypical ductal hyperplasia (ADH) have an increased risk of developing invasive ductal carcinoma (IDC) of breast. The importance of several molecular markers in breast cancer has been of considerable interest during recent years such as p53 and estrogen receptor alpha (ERα). However, p53 nuclear accumulation and ERα expression have not been assessed in ductal hyperplasia co-existing with ductal carcinoma in situ (DCIS) or IDC *versus *pure ductal hyperplasia without DCIS or IDC.

**Materials and methods:**

We investigated p53 nuclear accumulation and ERα expression in breast ductal hyperplasia in a cohort of 215 Chinese women by immunohistochemistry (IHC), which included 129 cases of pure ductal hyperplasia, 86 cases of ductal hyperplasia co-existing with DCIS (41 cases) or IDC (45 cases).

**Results:**

Nuclear p53 accumulation was identified in 22.8% of ADH (31/136), 41.5% of DCIS (17/41) and 42.2% of IDC (19/45), and no case of UDH (0/79). No difference in nuclear p53 accumulation was observed between pure ADH and ADH co-existing with DCIS (ADH/DCIS) or IDC (ADH/IDC) (*P *> 0.05). The positive rate of ERα expression was lower in ADH (118/136, 86.8%) than that in UDH (79/79, 100%) (*P *< 0.001), but higher than that in DCIS (28/41, 68.3%) or IDC (26/45, 57.8%) respectively (*P *< 0.001). The frequency of ERα expression was lower in ADH/DCIS (23/29, 79.31%) and ADH/IDC (23/30, 76.67%) than that in pure ADH (72/77, 93.51%) respectively (*P *< 0.05). There was a negative weak correlation between p53 nuclear accumulation and ERα expression as for ADH (coefficient correlation -0.51; *P *< 0.001).

**Conclusions:**

Different pathological types of ductal hyperplasia of breast are accompanied by diversity in patterns of nuclear p53 accumulation and ERα expression. At least some pure ADH is molecularly distinct from ADH/CIS or ADH/IDC which indicated the two types of ADH are molecularly distinct entities although they have the same morphological appearance.

## Introduction

Worldwide, breast cancer comprises 10.4% of cancer incidence among women, making it the second most common type of non-skin cancer (after lung cancer) and the fifth most common cause of cancer death [1]. In the last two decades, the incidence and mortality of breast cancer have climbed sharply in China, thus attracting increased attention of researchers [2]. Historically, beast cancer emerges by a multistep process which can be broadly equated to transformation of normal cells *via *the steps of hyperplasia, premalignant lesions and in situ carcinoma, invasive carcinoma which supported by evidences from clinical, pathological, and genetic studies [3-5]. It is a heterogeneous disease that encompasses a wide range of pathological entities and clinical behaviors, thus posing great challenges in understanding the precise molecular mechanisms of breast carcinogenesis [3]. Recent studies show that about 8% to 9% of women with benign lesions will be subsequently developed into invasive breast cancer [6,7]. It is quite unclear how invasive breast cancer develops through these ductal hyperplasias, which include usual ductal hyperplasia (UDH) and atypical ductal hyperplasia (ADH) [8].

The importance of some molecular markers in breast cancer has been of considerable interest during recent years, not only as prognostic markers, but also as predictors of response to therapy. p53 is the primary arbiter of the mammalian cells' response to stress. In its normal form, p53 can be involved in the induction of apoptosis and thus has a regulatory function over the cell cycle. In its mutant form, p53 inhibits apoptosis, loses control on cell cycle progression and thus helps tumor formation [9]. Nuclear p53 accumulation which associates with p53 mutation is one of the most common events during breast carcinogenesis [10-12]. Epidemiological and experimental evidences implicated oestrogens in the aetiology of breast cancer [13-17]. The biological actions of estrogens are mediated by binding to one of two specific estrogen receptors (ERs), ERα or ERβ, which belong to a family of ligand-regulated transcription factors [18]. ERα has been widely accepted as a prognostic marker and a predictor for endocrine therapy response of breast cancer [19,20]. In general, ERα-negative breast cancers are more aggressive and unresponsive to antiestrogens [21]. However, p53 nuclear accumulation and ERα expression have not been assessed in ductal hyperplasia co-existing with ductal carcinoma in situ (DCIS) or invasive ductal carcinoma (IDC) *versus *pure ductal hyperplasia without DCIS or IDC. The aims of this study were: (a) to assess p53 nuclear accumulation and ERα expression in pure ductal hyperplasia and ductal hyperplasia co-existing with DCIS or IDC; (b) to explore if there is a differential expression pattern of ERα and p53 nuclear accumulation between pure ductal hyperplasia and ductal hyperplasia co-existing with DCIS or IDC.

## Materials and methods

***Patients and tissues: ***129 cases of pure ductal hyperplasia of breast, 86 cases of ductal hyperplasia co-existing with DCIS (41 cases) and IDC (45 cases) were collected from surgical samples of women at the First Affiliated Hospital of China Medical University between 2005 and 2010. None of patients undergo chemotherapy, radiotherapy or adjuvant treatment before operation. Patients' ages ranged from 21 to 82, with an average age of 43.8 years old. Each case was reviewed independently by 2 pathologists (Chui-feng Fan and Min Song) with a subspecialty focus in breast pathology, and only those cases that both pathologists finally reached the unanimous diagnosis were used. In case of insufficient or unattainable material, original tissue blocks were reprocessed and new slides were created. The pathological types of breast ductal hyperplasia lesions have been classified according to WHO's criteria which published by Tavassoli FA et al [22]. All sections were reviewed for a comprehensive list of pathologic features, including margins (close margins were defined as tissue-free margins < 1 mm), the presence of concomitant UDH, ADH, DCIS and IDC. The pathological types of breast ductal hyperplasia lesions were summarized in Table 1. The cases of breast ductal hyperplasia lesions include 79 cases of UDH and 136 cases of ADH (16 cases of ductal intraepithelial neoplasia 1A (DIN 1A) and 120 cases of ductal intraepithelial neoplasia 1B (DIN 1B)). The study was approved by the regional ethics committee at China Medical University.

**Table 1:** Breast ductal hyperplasia lesions of the different pathological types

	Pure type	With DCIS	With IDC	Total
UDH	52	12	15	79
ADH	77	29	30	136
DIN 1A	1	9	6	16
DIN 1B	76	20	24	120
Total	129	41	45	215

***Immunohistochemistry: ***Formalin-fixed and paraffin-embedded specimens were cut into 4 μm-thick sections, which were subsequently de-waxed and hydrated. Immunohistochemical staining for ERα (sc-542, Santa Cruz, 1:200) and p53 (sc-47698, Santa Cruz, 1:100) were performed using UltraSensitive™ S-P kits (Maixin-Bio; P.R. China) according to the manufacturer's instructions and using the reagent supplied within the kit. For the negative control, phosphate-buffered saline (PBS) was used in place of the primary antibodies. We also adopted the German semi-quantitative scoring system in considering the staining intensity and area extent, which has been widely accepted and used in previous studies [23-25]. Every lesions was given a score according to the intensity of the nucleic staining (no staining = 0, weak staining = 1, moderate staining = 2, strong staining = 3) and the extent of stained cells (0% = 0, 1-10% = 1, 11-50% = 2, 51-80% = 3, 81-100% = 4; negative means 0% area staining, focally positive means 1-80% area staining, diffusely positive means 81-100% area staining). The final immunoreactive score was determined by multiplying the intensity scores with the extent of positivity scores of stained cells, with the minimum score of 0 and a maximum score of 12 [24-26]. Slides were independently examined by 2 pathologists (Chui-feng Fan and Min Song) as previously mentioned; however, if there was a discrepancy in individual scores both pathologists reevaluated together by reaching a consensus agreement before combining the individual scores. To obtained statistical results, a final score equal to or less than 1 was considered as negative, while scores of 2 or more were considered as positive.

***Statistical analysis: ***The results were evaluated using the χ2 test. The correlation between p53 nuclear accumulation and ERα expression was tested by using the Pearson chi-square test. All statistical analyses were performed using SPSS 13.0 for Windows (SPSS Inc., Chicago, IL, USA). Statistical significance in this study was set at P < 0.05. All reported P values are two-sided.

## Results

### p53 nuclear accumulation in ductal hyperplasia of breast

**Figure 1:**
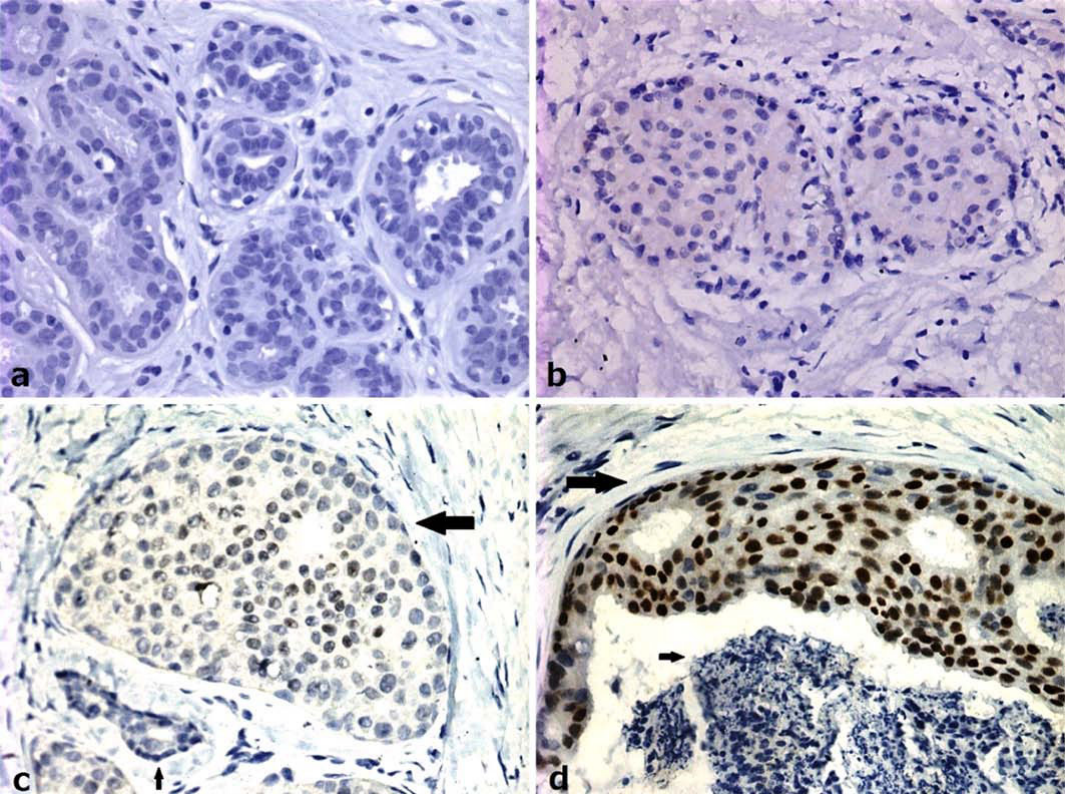
**Immunohistochemical staining of noninvasive breast lesions with antibody against p53**. p53 nuclear accumulation was not found in epithelial cells of normal ducts (a) and usual ductal hyperplasia (b) of breast. p53 positive staining in atypical ductal hyperplasia (c): the bigger arrow shows a breast duct filled with cells with atypical hyperplasia. The cells are quite identical in size and shape. Staining of p53 is seen in some nuclears (> 10%). The little arrow shows a normal duct without p53 nuclear accumulation. p53 positive staining in ductal carcinoma in situ (d): the bigger arrow shows a ductal carcinoma in situ with positive staining of p53 in nuclears (> 10%). The little arrow shows necrosis in the ductal carcinoma in situ. (× 40)

The phenotypic expression patterns of p53 in breast ductal hyperplasia were shown in Figure 1. Table 2 showed p53 nuclear accumulation in ductal hyperplasia of breast. No p53 nuclear accumulation was found in UDH (0/79) regardless of co-existing DCIS or IDC. p53 nuclear accumulation was detectable in 22.8% of ADH (31/136), higher than that in UDH (*P *< 0.001), lower than that in DCIS (41.5%, 17/41) or in IDC (42.2%, 19/45) respectively (*P *< 0.01). No difference in nuclear p53 accumulation were observed between pure ADH (14/77) and ADH/DCIS (9/29) (18.2% vs. 31.0%, *P *> 0.05) or ADH/IDC (8/30) (18.2% vs. 26.7%, *P *> 0.05).

**Table 2:** p53 nuclear accumulation and ERα expression in ductal hyperplasia of breast

	**Total no**.	p53 nuclear accumulation		*P-value*	ERα expression		*P-value*
					
		+	-		+	-	
UDH							
Pure type	52	0	52	*> 0.05*	52	0	*> 0.05*
With DCIS	12	0	12		12	0	
With IDC	15	0	15		15	0	
ADH							
Pure type	77	14	63	*> 0.05*	72	5	*<0.05*
With DCIS	29	9	20	*χ2 = 2.31*	23	6	*χ2 = 7.12*
With IDC	30	8	22		23	7	
DCIS							
With UDH	12	5	7	*> 0.05*	8	4	*> 0.05*
With ADH	29	12	17	*χ2 = 0.00*	20	9	*χ2 = 0.00*
IDC							
With UDH	15	7	8	*> 0.05*	11	4	*> 0.05*
With ADH	30	12	18	*χ2 = 0.18*	15	15	*χ2 = 1.38*

### ERα expression in ductal hyperplasia of breast

**Figure 2:**
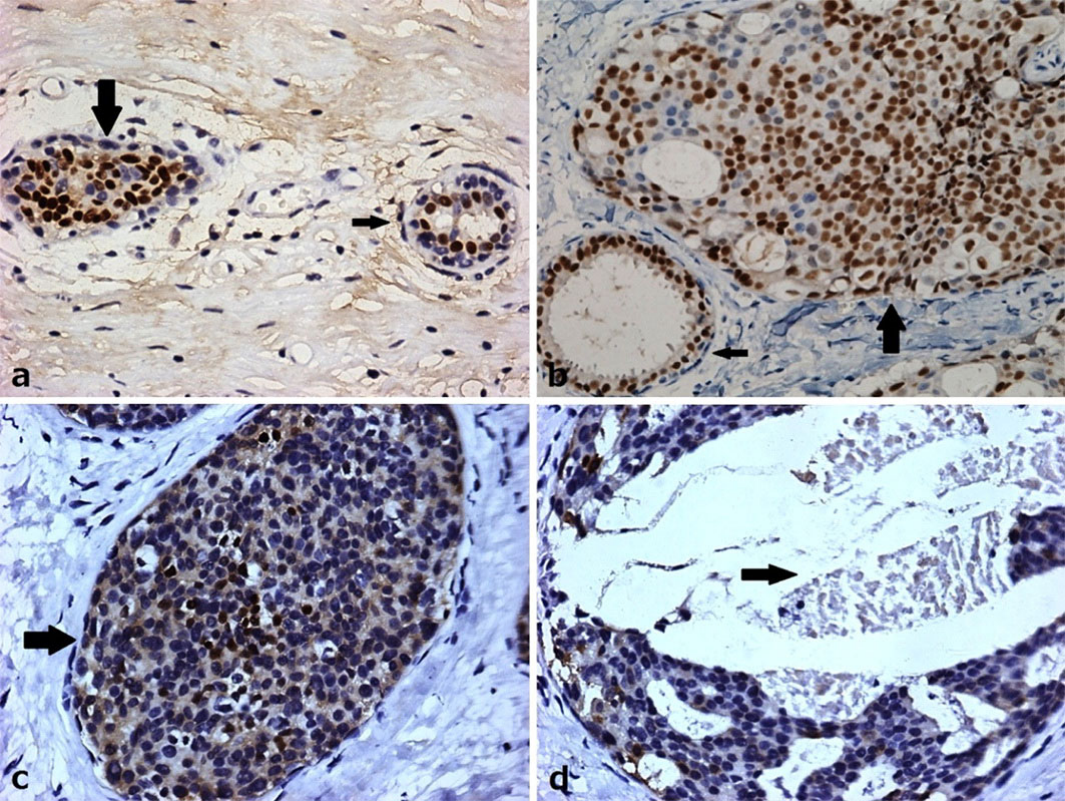
**ERα expression in noninvasive breast lesions**. a: ERα staining in epithelial cells of normal ducts (smaller arrow) and usual ductal hyperplasia (bigger arrow) of breast was located in nuclear. b: ERα staining was seen in all epithelial cells of a normal duct (smaller arrow) but was reduced in cells in a co-existing duct with atypical ductal hyperplasia (bigger arrow). c: The arrow shows a breast duct with atypical ductal hyperplasia with positive staining of ERα (> 10%) which was absent in some cells. d: ERα staining in a ductal carcinoma in situ was negative (< 10%). The arrow shows the necrosis. (× 40)

The phenotypic expression patterns of ERα protein in breast ductal hyperplasia were shown in Figure 2. The positive rate of ERα expression in breast ductal hyperplasia was summarized in Table 2.The positive rate of ERα expression was lower in ADH (118/136, 86.8%) than that in UDH (79/79, 100%) (*P *< 0.001), but higher than that in DCIS (28/41, 68.3%) or IDC (26/45, 57.8%) respectively (*P *< 0.001). The frequency of ERα expression was lower in ADH/DCIS (23/29, 79.31%) and ADH/IDC (23/30, 76.67%) than that in pure ADH (72/77, 93.51%) respectively (*P *< 0.05).

### Correlation between p53 nuclear accumulation and ERα expression

**Figure 3:**
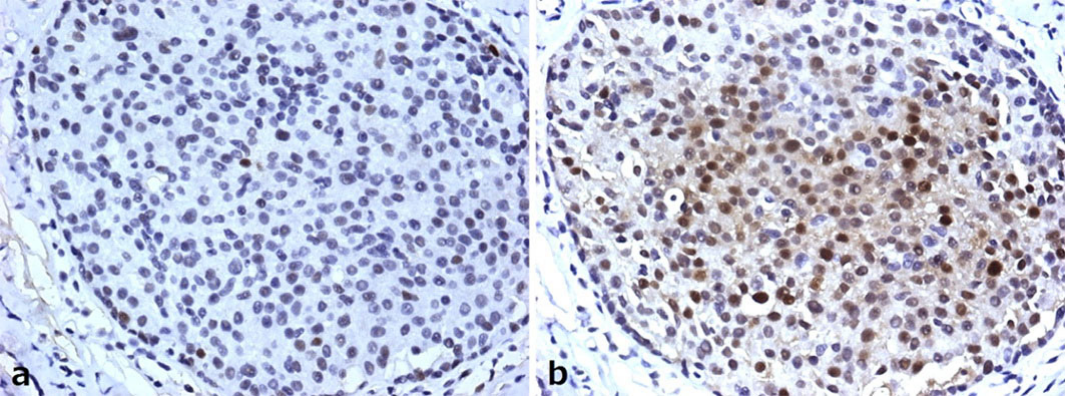
**A case of ADH of breast with concurrent increased p53 nuclear accumulation (a) and reduced ERα expression**. There were some cells (> 10%) with weak p53 staining in a. While some cells (> 10%) were absent of ERα staining in b.

There was no correlation between p53 nuclear accumulation and ERα expression in any type of ductal hyperplasia of breast (*P *> 0.05). But as shown in Figure 3. p53 nuclear accumulation and ERα expression had inverse patterns of alterations in ADH of breast. As for ADH, which shown in Table 3 the correlation coefficient was -0.512 between p53 nuclear accumulation and ERα expression (*P *< 0.001).

**Table 3:** Correlation of p53 nuclear accumulation with ER? expression in ADH

	p53 unclear accumulation		
		
	+	-	
ERα expression +	17	101	*r *= -0.512
ERα expression -	14	4	*P *< 0.001

Correlation between p53 nuclear accumulation and ERα expression; *r *= correlation coefficient (*n *= 136).

## Discussion

p53 is located on human chromosome 17p and its encoding protein mediates its tumor suppressor function *via *the transcriptional regulation or repression of various genes [26-29]. p53 had been suggested to be predictive of risk for subsequent breast carcinogenesis, p53 nuclear accumulation has been identified as a poor prognostic marker in breast cancer [30]. The immunohistochemical detection of nuclear p53 protein accumulation is highly associated with p53 gene mutations in breast cancer tissues [31], in benign breast lesions it has been associated with elevated risk of progression to breast cancer [32]. In this study, we found nuclear p53 accumulation occurred in ADH but not in UDH regardless of co-existing DCIS or IDC. Nuclear p53 accumulation was not significantly different between pure ADH and ADH co-existing DCIS or IDC. It was in accordance with previous studies that UDH was considered to represent a benign proliferation of ductal epithelial cells, whereas ADH represents the first clonal neoplastic expansion of these cells [33]. It is clear that not all ADH will progress into DCIS or IDC during the patient's lifetime. However, we found no differences in p53 expression between pure ADH and ADH co-existing with DCIS or IDC. Maybe there are more molecular alteration counteracts with p53 or p53 itself is an initiative factor in breast carcinogenesis.

Epidemiological and experimental evidences implicated estrogens in the aetiology of breast cancer which play a central role in the growth and differentiation of normal breast epithelium [13-17]. ERα status has also been shown to have prognostic value in breast cancer, although the importance of hormone-receptor status lies rather as a predictor of response to endocrine therapy. A potential mechanism of hormone resistance is the acquired loss of ERα gene expression at the transcriptional level during breast carcinogenesis [34-37]. Here, we found ERα expression in all UDH regardless of co-existing DCIS or IDC though there were occasionally sporadic staining patterns, and there was significant loss of ERα expression in ADH and breast carcinoma, ERα was decreasingly expressed from UDH to ADH, DCIS or IDC. Our findings support that UDH and ADH are different ductal hyperplasia lesions of breast, they have pathological types which accompanied by diversity in pattern of genetic expression.

In our study, a significant difference in ERα expression was found between pure type ADH and ADH/DCIS or ADH/IDC, suggested that the subsets of ADH/CIS or ADH/IDC may have different molecular genetics in comparison with the pure ADH without DCIS or IDC. ADH and ADH/DCIS or ADH/IDC have similar morphology, but have different ERα expression. Furthermore, we found a negative weak correlation between p53 nuclear accumulation and ERα expression as for ADH (coefficient correlation -0.512; *P *< 0.001). Experiments in vitro suggested that ERα opposes p53-mediated apoptosis in breast cancer cells by Sayeed A [38]. Shirley SH performed animal experiments to show that p53 genotype was correlated with ER expression and response to tamoxifen in mammary tumors arising in mouse mammary tumor virus-Wnt-1 transgenic mice. They changed the p53 expression of MCF-7 cells with doxorubicin or ionizing radiation, ER expression was also changed. In MCF-7 transfected with WT p53, transcription from the ER promoter was increased 8-fold, they concluded that p53 may regulate ER expression [39]. Based on our study, further investigation about the relation between p53 and ER appear to be warranted in breast carcinogenesis.

ERα loss in breast carcinoma is considered an unfavorable factor for patients partly due to the accordingly reduced sensitivity of cancer cells to endocrine therapy. There are patients with ERα (-) breast carcinomas but has ERα ( + ) surrounding breast tissues including those have atypical hyperplasia. These patients are often not supposed to be given the endocrine therapy. But what the ERα ( + ) surrounding breast tissues means to the endocrine therapy protocol is still mysterious and intriguing. Based on our study, ERα loss may be partly due to p53 accumulation during carcinogenesis of breast carcinoma. On the other hand there also may be some other unknown molecules involved in the interplays with ERα loss instead of p53 nuclear accumulation. To restore the ERα ( + ) phenotype of breast carcinogenesis for better outcome of endocrine therapy, further investigation of molecules involved is necessary.

In summary, we found the diversity of the pathological type is accompanied by diversity in pattern of genetic expression. And at least some pure ADH is molecularly distinct from ADH/CIS or ADH/IDC which indicated the two types of ADH are molecularly distinct entities although they have the same morphological appearance. Molecular differences between pure and synchronous lesions support re-evaluation of current models of breast cancer initiation, progression, and risk.

## Competing interests

The authors declare that they have no competing interests.

## Authors' contributions

JF and MXY designed the research and wrote the paper. MXY and FCF collected the breast lesion tissues and carried out experiments. WJ, ZHC and YF analyzed the data. All authors have read and approved the manuscript.
